# Priority of PET-CT vs CT Thorax for EBUS-TBNA 22G vs 19G: Mesothorax Lymphadenopathy

**DOI:** 10.7150/jca.59892

**Published:** 2021-08-05

**Authors:** Paul Zarogoulidis, Haidong Huang, Zhenli Hu, Ning Wu, Jiannan Wang, Dimitris Petridis, Kosmas Tsakiridis, Dimitris Matthaios, Christoforos Kosmidis, Wolfgang Hohenforst-Schmidt, Christos Tolis, Ioannis Boukovinas, Nikolaos Courcoutsakis, George Nikolaidis, Chrysanthi Sardeli, Chong Bai, Chrysanthi Karapantzou

**Affiliations:** 1 Pulmonary Department, “Bioclinic” Private Hospital, Thessaloniki, Greece.; 2 3 rd University General Hospital, “AHEPA” University Hospital, Thessaloniki, Greece.; 3 Department of Respiratory & Critical Care Medicine, Changhai Hospital, The Second Military Medical University, Shanghai, China.; 4 Department of Food Technology, School of Food Technology and Nutrition, Alexander Technological Educational Institute, Thessaloniki, Greece.; 5 Thoracic Surgery Department, “Interbalkan” European Medical Center, Thessaloniki, Greece.; 6 Oncology Department, General Hospital of Rhodos, Rhodos, Greece.; 7 Sana Clinic Group Franken, Department of Cardiology/Pulmonology/Intensive Care/Nephrology, “Hof” Clinics, University of Erlangen, Hof, Germany.; 8 Private Oncology Cabinet, Ioannina, Greece.; 9 Oncology Department, “Bioclinic” Private Hospital, Thessaloniki, Greece.; 10 Radiology Department, University General Hospital of Alexandroupolis, Democritus University of Thrace, Alexandroupolis, Greece.; 11 Surgery Department, “General Clinic Euromedica” Private Clinic, Thessaloniki, Greece.; 12 Ear, Nose and Throat (ENT) Department, Ludwig-Maximilians University of Munich, Munich, Germany.; 13 Department of Pharmacology & Clinical Pharmacology, School of Medicine, Faculty of Health Sciences, Aristotle University of Thessaloniki, Thessaloniki, Greece.

**Keywords:** EBUS-TBNA, lung cancer, lymphoma, 22G needle, 19G needle, CT, PET-CT, Mesothorax

## Abstract

**Introduction:** Lung lesions and undiagnosed mesothorax lymphadenopathy is an issue that several doctors face in the everyday clinical practice. PET-CT and CT of the thorax are usually the first examinations to identify characteristics of the lesions before biopsy.

**Patients and Methods:** We performed a retrospective study with 450 patients that had EBUS-TBNA with 22G, Upgraded 22G and 19G needles with and without PET-CT in order to identify the cost effeteness of performing EBUS-TBNA before or after PET-CT. All centers used the same PET-CT equipment and EBUS-TBNA system. Three types of needle were used for the endoscopy in order to identify similarities and differences for the cost-effectiveness. The costs in every center for every examination and materials were the same.

**Results:** There were more block slices for 19G>22Gupgraded>21G>22G and there was cost-effectiveness when in general PET-CT was performed prior to biopsy of any lesion. 19G needle was more effective for lymphomas, while 22Gupgraded and 21G needles were more cost-effective when used for smaller lesions for primary lung cancer of metastatic disease.

**Conclusions:** We have been using PET-CT and EBUS-TBNA in the everyday clinical practice according to the current guidelines for initial disease staging and re-staging. However; we can also use both in a cost effective method based on the initial radiologic findings.

## Introduction

Several patients are admitted to hospitals with different symptoms and radiology exams often reveal lesions in the thorax. Computed tomography (CT) of the thorax with or without intravenous contrast can be easily used in every tertiary hospital. Positron emission tomography (PET-CT) has been added in our diagnostic algorithm the last 12 years. It has been evaluated for several diseases both for benign and malignant diseases. It can be used either for initial diagnosis or initial staging, but also for the restaging of non-small cell lung cancer (NSCLC) disease 1-5. PET-CT has a substantial diagnostic value for single nodules of ≥0.8 mm. Several centers take a delayed second examination 20-30 minutes after the initial examination in order to identify malignancies with low metabolic rate. However; there are still issues where malignancies cannot be identified to the low metabolic rate 6. There is also still an issue with benign disease such as; sarcoidosis where PET-CT is under evaluation as a diagnostic tool 7. Endobronchial ultrasound (convex) is an endoscopic tool again with more than 10 years in service with 22G, 21G and 19G needles for biopsy. It is an excellent tool for initial diagnosis and staging in different diseases 8-10. The sample size is enough to perform gene investigation for several cancer types 11, 12. The role of PET-CT and EBUS-TBNA has been for several malignancies and an algorithm has been set. However; we do not have an algorithm to follow for undiagnosed mesothorax lymphadenopathy. Should we first perform PET-CT or we can proceed directly to biopsy. There is the issue of cost-effect regarding the needle that we have to use and the unnecessary PET-CT examinations which have a high cost. We performed a retrospective study to identify an algorithm through several institutions following the same diagnostic protocol and examination costs.

## Patients and Methods

We enrolled 450 patients from our oncology and pulmonary outpatient cabinets. These patients came to our cabinets either with a CT of the thorax performed with intravenous contrast or PET-CT. The investigational review board (IRB) of the 3^rd^ University General Hospital, “AHEPA” University Hospital, Thessaloniki, Greece provided us with the authorization of our study. A CT of the thorax with contrast costs 160 euros and a PET-CT 1150 euros. All patients had mesothorax lymphadenopathy. Endobronchial ultrasound (convex probe) was performed with a PENTAX EB-1970UK, EPK-1000 video processor and a Hitachi EUB-7500HV ultrasound source. The following needles were used; a) 22G Mediglobe^®^, b) 22G upgraded Mediglobe^®^, c) 21G Olympus^®^ and d) 19G Olympus^®^. The costs for the needles were as follows: a) 170 euros, b) 330 euros, c) 440 euros and d) 520 euros. The EBUS procedure without the needles had a cost of 1300euros in every department including anesthesiology drugs, medical staff (nurse, anesthesiologist and doctor), pathology result and all other materials necessary for the examination. There was an extra cost of 350 euros for next generation sequencing (NGS) when this was necessary, but this was not information included in our study. Patients that had before the EBUS a PET-CT their examination had a total cost as follows for each needle: a) 259.475 euros, b) 280.780 euros, c) 289.000 euros and d) 112860 euros. All 344 patients that had an initial PET-CT before EBUS had a diagnosis and no false negative result. The rest of the 106 patients had only a CT of the thorax with intravenous contrast and the cost of the examination was as follows for each needle: a) 63.570 euros, b) 41.170 euros, c) 64.600 euros and d) 15.840 euros. All 106 patients did not have a diagnosis, and PET-CT was performed and then re-biopsy with EBUS-TBNA with the same needles in different puncture point where the PET-CT was highly positive with an SUV ≥3. The cost for the new examination of these patients after the PET-CT was as follows: a) 108.420 euros, b) 67.620 euros, c) 103.700 euros and d) 25.040 euros. The time between the new re-biopsy was 15 days for each patient. The money loss in total for the 106 patients was 304.780 euros. The total cost of the 344 patients was 942.115 euros (**Table 1**).

### Statistics

#### Methods

The efficiency of the positron emission tomography (PET) was investigated on patients with different cancer types using different needle sizes and dissecting various slices. The number of slices as affected by the needle size was detected using the 95% confidence interval plot of means based on ANOVA's error mean square. Also, the association among needle size, lesion size and cancer type was investigated employing a multiple correspondence analysis.

The efficiency of the Modified electric convulsive treatment (MECT meaning patients that had PET-CT before the EBUS biopsy), was detected by plotting the slices mean against the lesion size and cancer type as similarly described above.

## Results

**Figure 1:**
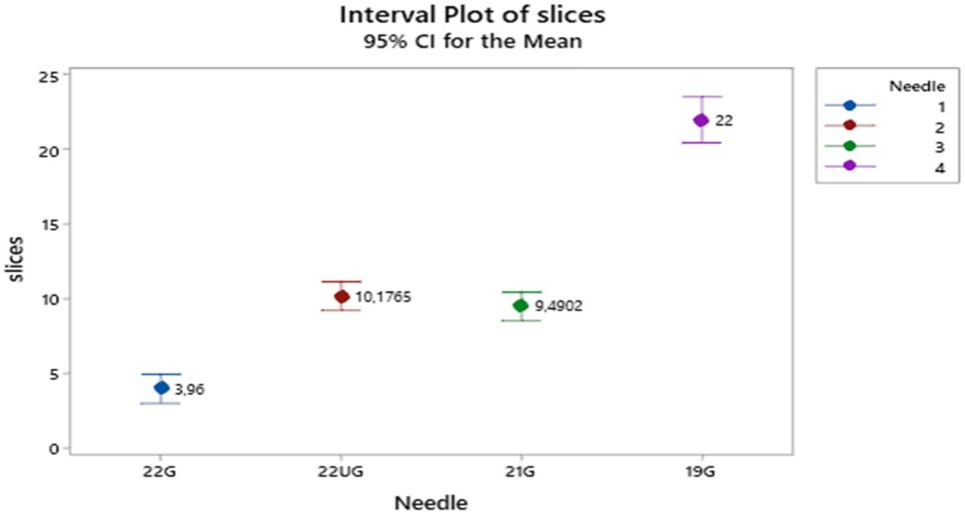
Mean number of slices distribution according to needle types. Vertical lines denote the 95% confidence intervals of means based on the ANOVA's error mean square. Levels means whose intervals do not overall differ significantly. The pooled standard deviation is used to calculate the intervals.

For the PET apparatus **Figure 1** shows that the mean number of slices is twice much higher when needle 19 is used than those affiliated with the needles (22U) 22G upgraded Mediglobe^®^ and 21G (equally performing) and the latter are again twice much higher than the mean slices associated with needle 22G (170 euros needle Mediglobe^®^).

**Figure 2:**
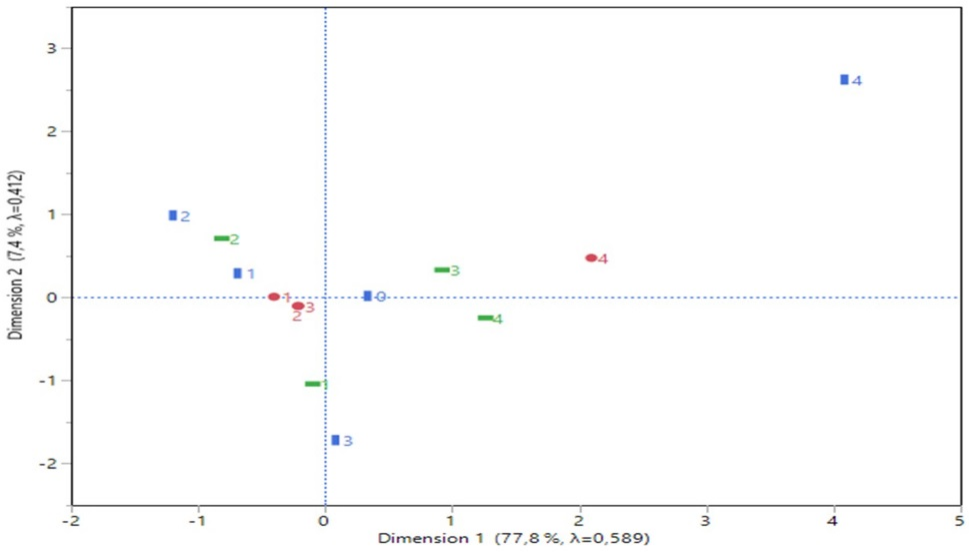
Statistical output of a correspondence analysis: a two dimensions plot, the Greenacre adjusted inertia (percentage contribution to the first two dimensions) and partial contributions of coordinates to each dimension.

The multiple correspondence analysis pointed out an 85.25% explanation of inertia variation (**Figure 2**) concerning the first two dimensions.

Dimension 1 is best described by needle type 4 (19G Olympus^®^ and cancer type 4 (Hodgkin) (see the partial contributions of coordinates in **Figure 2**), both arranged at the right part of corresponding plot and far apart from the rest categories. Similarly, dimension 2 was best formed by lesion size 1 (≥1 cm) and cancer type 3 (higher contribution of the coordinates-Non-Hodgkin), both positioned at the lower part of the plot and also far apart from the other categories. Cancer type 2 (metastatic from other organs than the thorax) and lesion size 2 (≥2 cm) are also affiliated each other due to their close distance and moderate contributions for the axes formation.

**Figure 3:**
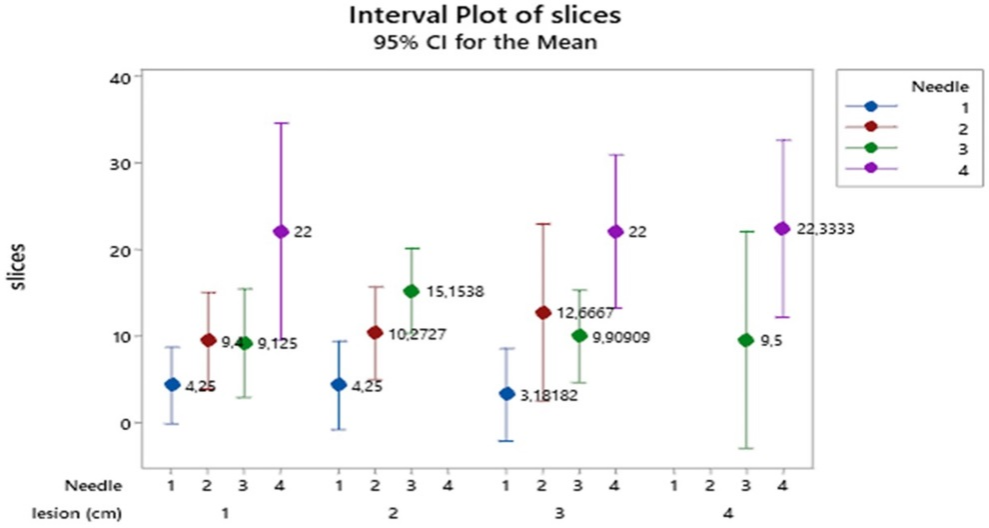
Mean number of slices distribution according to the combined effect of needle types and lesion size. Vertical lines denote the 95% confidence intervals of means based on the ANOVA's error mean square. Levels means whose intervals do not overall differ significantly. The pooled standard deviation was used to calculate the intervals.

For the MECT (MECT meaning patients that had PET-CT before the EBUS biopsy) apparatus, the 95% confidence interval plot of slice means (**Figure 3**) indicate that needles size 4 (19G Olympus^®^) produce the highest number of slices, those of size 2 (22G upgraded Mediglobe^®^) and 3 (21G Olympus^®^) intermediate ones and those of size 1 (≥1 cm) the lowest values (absent for lesion 4 ≥4 cm). Lesions show a similar pattern of slices in the needles across all lesion categories.

The value of slices, needle type and cancer type in fact present a proportional cost-effectiveness due to the following reasons:

- The higher the number of slices, the larger the needle, the higher the cost;
- The cancer type Hodgkin, non-Hodgkin vs the other cancer types, increases the operation cost;
- Concerning the lesion size no cost-effective is reasonably expected thus, no real statistical information is possible to support an apparent effect as explained by the pre-explained reasons.

## Discussion

**Figure 4:**
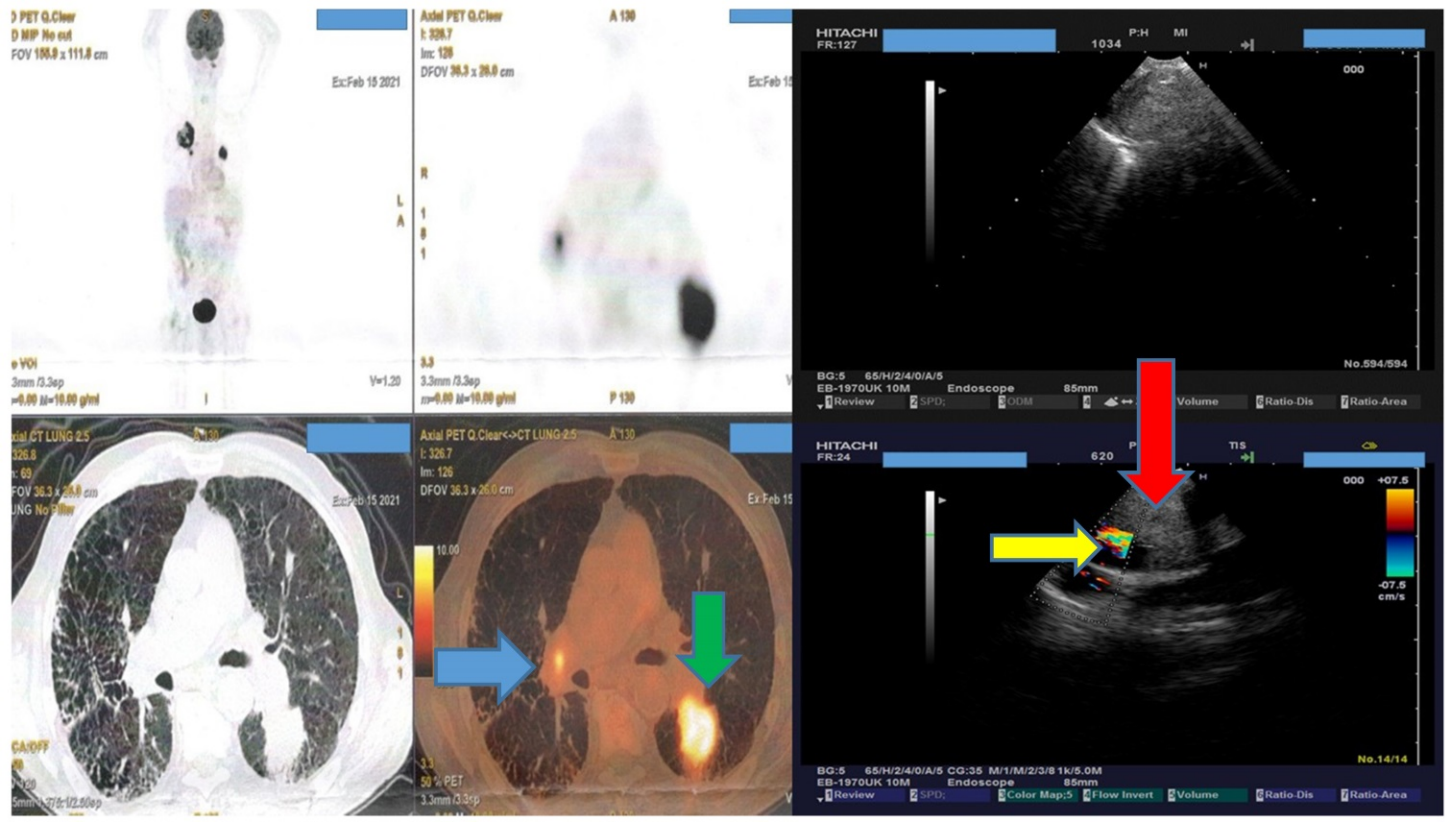
Lymphnode 11L does not have any SUV uptake. Blue arrow indicates a positive lymphnode station L10R, green arrow indicates a lung cancer mass, again red arrow indicates the same lymphnode station on the left (L10R with blue arrow) and red arrow indicates a right pulmonary artery branch next to lymphnode station L10R.

Several studies have compared the cost-effectiveness and safety of EBUS-TBNA for diagnosis versus mediastinoscopy 13, 14. Certainly according to current guidelines for lung cancer PET-CT and EBUS-TBNA have an algorithm before mediastiscopy is performed 15. However; several patients refer to internal medicine doctors and general practitioners and not always to oncologists and pulmonary physicians. Currently there are no guidelines for undiagnosed lymphadenopathy of mesothorax. All our patients had malignancy because an initial work-up with medical history and because of the expertise of our departments patients referred to us were most likely to have malignancy. In other centers a part of these patients would include benign disease such post infection lymphadenopathy or sarcoidosis. We observed in our study again as it has been previously identified that 19G needle has larger biopsy sample almost double the size of a 22G needle 8, 16. The biopsy sample size was as follows in our study; d>c=b>a based on the needles. This observation was made with the number of cell block slices that were produced from each biopsy. The sample size did not differ among the different lesion size, this is due to the ebus technique where we have real time visualization of the lesion and several punctures can be made with safety indifferent of the needle. Needle size 19G we know from previous studies that is efficient for lymphomas 17. Cost-effectiveness is an issue for every health system and this is decided for each patient upon admission. A PET-CT is necessary to identify necrosis and the intensity of the lesions. Also, we can identify the part of the lesion which has the highest intensity in order to puncture this specific site (**Figure 4**).

We can observe in **Figure 4** that although someone could puncture lymphnode number 11L in this patient, no result would be available because there is no SUV uptake. This information would not available with a CT of the thorax even with i.v contrast.

**Figure 5:**
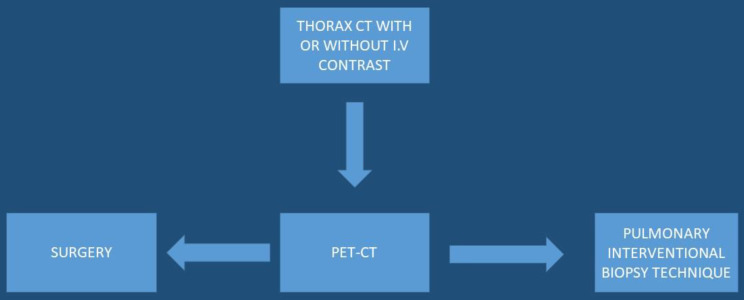
Upon lung mass identification (with or without lymphadenopathy) from a thorax-CT, PET-CT should be directly performed,

Several studies have been performed where elastography was used to identify the strain ratio of the lesions, however; without efficient results 18-20. Moreover; the use of the needle is another important cost, if we have the same number of block slices with less expenses then it would be preferred. However; health systems work different between Europe, China and the United States, and certainly PET-CT or EBUS-TBNA is not available in every hospital. The most important cost-effective decision is finally made by the initial physician and it would be preferred that these patients should be directed to centers of expertise with malignancies. A cost-effectiveness algorithm should be proposed by the scientific societies for the diagnostic work, not only for oncologists and pulmonary physicians, but also for other specialties (**Figure 5**).

## Figures and Tables

**Table 1:** Cost-Effectiveness of biopsies

NEEDLE	22G MEDIGLOBE®	22G UPGRADED MEDIGLOBE®	21G OLYMPUS®	19G OLYMPUS®
COST IN EUROS PER UNIT	170	330	440	520
344 PATIENTS WITH PET-CT	259.475	280.780	289.000	112.860
106 PATIENTS WITH CT THORAX	63.570	41.170	64.600	15.840
106 WITH RE-BIOPSY AFTER PET-CT	108.420	67.620	103.700	25.040
